# Predicting Openness of Communication in Families With Hereditary Breast and Ovarian Cancer Syndrome: Natural Language Processing Analysis

**DOI:** 10.2196/38399

**Published:** 2023-01-19

**Authors:** Vasiliki Baroutsou, Rodrigo Cerqueira Gonzalez Pena, Reka Schweighoffer, Maria Caiata-Zufferey, Sue Kim, Sharlene Hesse-Biber, Florina M Ciorba, Gerhard Lauer, Maria Katapodi, CASCADE Consortium

**Affiliations:** 1 Department of Clinical Research University of Basel Basel Switzerland; 2 Center for Data Analytics University of Basel Basel Switzerland; 3 Competence Centre for Healthcare Practices and Policies Department of Business Economics, Health and Social Care University of Applied Sciences and Arts of Southern Switzerland Manno Switzerland; 4 College of Nursing Yonsei University Seoul Republic of Korea; 5 Department of Sociology Boston College Chestnut Hill, MA United States; 6 Department of Mathematics and Computer Science University of Basel Basel Switzerland; 7 Gutenberg-Institut für Weltliteratur und schriftorientierte Medien Abteilung Buchwissenschaft Johannes Gutenberg Universität Mainz Philosophicum Mainz Germany; 8 See Acknowledgments

**Keywords:** cascade testing, dictionary-based approach, family communication, hereditary breast and ovarian cancer, HBOC, sentiment analysis, text mining, natural language processing, cancer, hereditary

## Abstract

**Background:**

In health care research, patient-reported opinions are a critical element of personalized medicine and contribute to optimal health care delivery. The importance of integrating natural language processing (NLP) methods to extract patient-reported opinions has been gradually acknowledged over the past years. One form of NLP is sentiment analysis, which extracts and analyses information by detecting feelings (thoughts, emotions, attitudes, etc) behind words. Sentiment analysis has become particularly popular following the rise of digital interactions. However, NLP and sentiment analysis in the context of intrafamilial communication for genetic cancer risk is still unexplored. Due to privacy laws, intrafamilial communication is the main avenue to inform at-risk relatives about the pathogenic variant and the possibility of increased cancer risk.

**Objective:**

The study examined the role of sentiment in predicting openness of intrafamilial communication about genetic cancer risk associated with hereditary breast and ovarian cancer (HBOC) syndrome.

**Methods:**

We used narratives derived from 53 in-depth interviews with individuals from families that harbor pathogenic variants associated with HBOC: first, to quantify openness of communication about cancer risk, and second, to examine the role of sentiment in predicting openness of communication. The interviews were conducted between 2019 and 2021 in Switzerland and South Korea using the same interview guide. We used NLP to extract and quantify textual features to construct a handcrafted lexicon about interpersonal communication of genetic testing results and cancer risk associated with HBOC. Moreover, we examined the role of sentiment in predicting openness of communication using a stepwise linear regression model. To test model accuracy, we used a split-validation set. We measured the performance of the training and testing model using area under the curve, sensitivity, specificity, and root mean square error.

**Results:**

Higher “openness of communication” scores were associated with higher overall net sentiment score of the narrative, higher fear, being single, having nonacademic education, and higher informational support within the family. Our results demonstrate that NLP was highly effective in analyzing unstructured texts from individuals of different cultural and linguistic backgrounds and could also reliably predict a measure of “openness of communication” (area under the curve=0.72) in the context of genetic cancer risk associated with HBOC.

**Conclusions:**

Our study showed that NLP can facilitate assessment of openness of communication in individuals carrying a pathogenic variant associated with HBOC. Findings provided promising evidence that various features from narratives such as sentiment and fear are important predictors of interpersonal communication and self-disclosure in this context. Our approach is promising and can be expanded in the field of personalized medicine and technology-mediated communication.

## Introduction

Natural language processing (NLP) is a computer-assisted analytical approach for automatically evaluating and interpreting human language by extracting meaningful insights from textual data sets [1-3]. NLP has been broadly used in various fields in the recent past, for example, in financial and business marketing, education, and health care [4-8]. The typical applications of NLP include information extraction, sentiment and semantic analysis, text classification, and text summarization. Among the different NLP applications, sentiment analysis has become particularly popular in recent years following the rise of digital communication and social media [2,9]. Sentiment analysis aims to assess whether people’s opinions, emotions, and attitudes toward a certain event or experience are positive, negative, or neutral [3,10,11] and generates valuable insights that lead to the improvement of a new service or product.

In health care–related studies, patient-reported insights are an essential component of personalized medicine and contribute to optimal health care delivery. Researchers have applied NLP to extract and analyze patient-reported insights from social media and for different topics, for example, social exchange patterns in web-based health platforms [12], needs of patients and caregivers in different disease entities [13], online support groups for patients with breast cancer [14], or awareness for Lynch syndrome (LS) [15]. A major limitation of this approach is that population characteristics (age, socioeconomic status, etc) are often unavailable, which limits the clinical applicability of findings and may create disparities either due to increased representation or lack thereof of certain population subgroups. Others have applied NLP to clinical notes originating from electronic medical records to describe patients’ experiences with symptoms [16] or free-text data from patient surveys evaluating the quality of hospital services [17]. One limitation of this approach is the lack of depth in these data sources, either because they lack the patient’s perspective or because the texts are limited in scope and volume. We identified only a few studies that applied NLP to unstructured narratives collected from in-depth interviews aiming to describe experiences with cancer ambulatory services [18] or to predict changes in substance use [19] and perceived loneliness among older adults [20].

NLP and sentiment analysis in the context of intrafamilial communication for genetic cancer risk is unexplored. Due to privacy laws, individuals carrying pathogenic variants in cancer-causing genes have a key role in disseminating information to relatives and in advocating for genetic testing [21]. This self-disclosure process is currently the main avenue to alert relatives to their own risk of carrying the pathogenic variant. Self-disclosure is a process of interpersonal communication by which one person reveals information about themselves to another person, or a small intimate group, for example, their family. The information exchange can be based on verbal and nonverbal cues and can be face to face or technology mediated. Most importantly, in addition to information exchange, self-disclosure can include thoughts, emotional experiences and feelings, aspirations, goals, fears, likes, and dislikes [22]. During self-disclosure, humans adjust and adapt their verbal and nonverbal communication, and messages are produced, interpreted, understood, or misunderstood [23,24]. Intrafamilial communication for genetic cancer risk may involve significant levels of uncertainty and potential conflicts since the meaning of self-disclosure about the cancer-causing variant can be shaped by opposing arguments and negative responses from others. Indeed, information exchange about genetic cancer risk may be easier with some family members or may present a particularly difficult moment with others [25,26].

Predicting openness of communication and examining the role of sentiment in intrafamilial communication of genetic cancer risk may be used to enrich message tailoring in technology-assisted interventions. In this study, we examined the role of sentiment in predicting openness of communication about genetic cancer risk associated with hereditary breast and ovarian cancer (HBOC) syndrome. HBOC is a hereditary cancer syndrome that affects both men and women and accounts for a significant number of different cancers, such as breast, ovarian, pancreatic, and prostate [27]. Sharing information about HBOC-causing pathogenic variants is a complex process of intrafamilial communication and a key element of public health interventions aiming to promote cascade testing of relatives and cancer prevention and control [28,29]. In this study, we used narrative data collected with in-depth interviews: first, to quantify openness of communication about HBOC cancer risk, and second, to examine the role of sentiment in predicting openness of communication.

## Methods

### Design, Population, Settings, and Procedures

This analysis is part of a larger ongoing study, the Swiss CASCADE cohort, which follows adult (aged ≥18 years) men and women from families that harbor pathogenic variants associated with HBOC or LS. The cohort includes individuals who had genetic testing, confirming either the presence or the absence of the familial pathogenic variant, and their untested relatives with unknown mutation status. Eligible participants may have had a cancer diagnosis, or they could be cancer-free at the time of enrolment in the study. Recruitment takes place at 8 different oncology and genetic testing centers in the German-, French-, and Italian-speaking regions of Switzerland. The study collects survey data designed to elicit factors that enhance cascade genetic testing and cancer surveillance for HBOC and LS. A subsample of participants has consented to provide narrative data regarding family communication of test results. For the purposes of this paper, we focused only on individuals who have had genetic testing for HBOC-associated pathogenic variants and accepted to provide narrative data.

### Ethics Approval

The study protocol has been approved by the Ethics Committee of Northwest Switzerland (BASEC 2016-02052) and is publicly available (ClinicalTrials.gov NCT03124212) [30]. We also used available data from participants in the K-CASCADE study (ClinicalTrials.gov NCT04214210) in South Korea, which focuses on HBOC. K-CASCADE and the collaboration of the 2 studies has been approved by local ethics committees (Severance Hospital Institutional Review Board: 4-2020-0520). K-CASCADE is identical to the Swiss CASCADE in respect to scope, research design, participant eligibility criteria (except for age ≥19 years), and data collection methodology. Participants to K-CASCADE are recruited from 5 hospitals in South Korea [31].

### Narrative Data

Narrative data included in this paper were collected from 44 individuals living in Switzerland and 9 in South Korea. The in-depth interviews were conducted between April 2019 and June 2021 either face to face or online (after April 2020 due to the COVID-19 pandemic) by trained research staff in German, French, Italian, English, and Korean using the same interview guide. Interview questions were designed to explore general communication patterns within family networks and specific experiences and barriers of family communication regarding genetic risk including discussions with health care providers. Examples of questions included in the interview guide are “What are some issues (barriers) that people might experience, related to sharing genetic risk information with family members?” and “Think of your own experience of (not) sharing genetic risk information with family members. What did you do and how did you decide about it?” Interviews were recorded, and all narrative data were transcribed verbatim in the original language in Microsoft Word and translated into English for this paper.

### Survey Data

Survey data were collected on an ongoing basis, starting in fall 2017 and occurring approximately 18-24 months apart. Self-administered surveys assessed demographic and clinical characteristics [30]. The surveys also included investigator-developed items that have been associated with family communication and intention to inform relatives about genetic cancer risk. These items assess informational support among family members, preference for patient-mediated communication of genetic testing results, and perceived utility of genetic testing for relatives (Textbox 1). These items are scored on 7-point Likert-type scales ranging from 1 “Strongly Disagree” to 7 “Strongly Agree.” Respondents also completed the Informing Relatives Inventory (IRI), a 37-item scale assessing knowledge, motivation, and self-efficacy to disclose genetic cancer risk to relatives [32]. IRI items are also scored on a 7-point Likert-type scale, with higher overall score indicating greater intention to inform relatives about genetic cancer risk.

**Items from the CASCADE baseline survey used for this study.**

Demographic characteristics

- Age
- Sex (female/male)
- Education level (elementary-, high school–, or technical school–graduate or academic degree)
- Marital status (married or living as married, single, divorced or separated, or widowed)
- Employment status (working full time, nonworking, or retired)

Clinical characteristics

- Cancer status (affected or never diagnosed with cancer)
- Genetic testing result (positive or negative for the familial pathogenic variant)

Family communication

- “In our family when I have a health problem there is great willingness to share information with each other”
- “I would prefer not to discuss about genetic testing results with anyone in my family”
- “If you have blood relatives, would it be useful for them to have genetic testing?”

### Data Analysis Overview

First, we examined narratives to assess “openness of communicating” genetic test results and cancer risk with relatives and with health care providers. Second, we categorized the text of each narrative as describing either a positive or negative sentiment toward experiences with genetic testing and health care services. Third, we examined whether demographic and clinical characteristics and sentiment, as expressed in the narrative, can predict “openness of communicating” genetic risk from tested individuals to relatives.

### NLP Model Development

**Figure 1:**
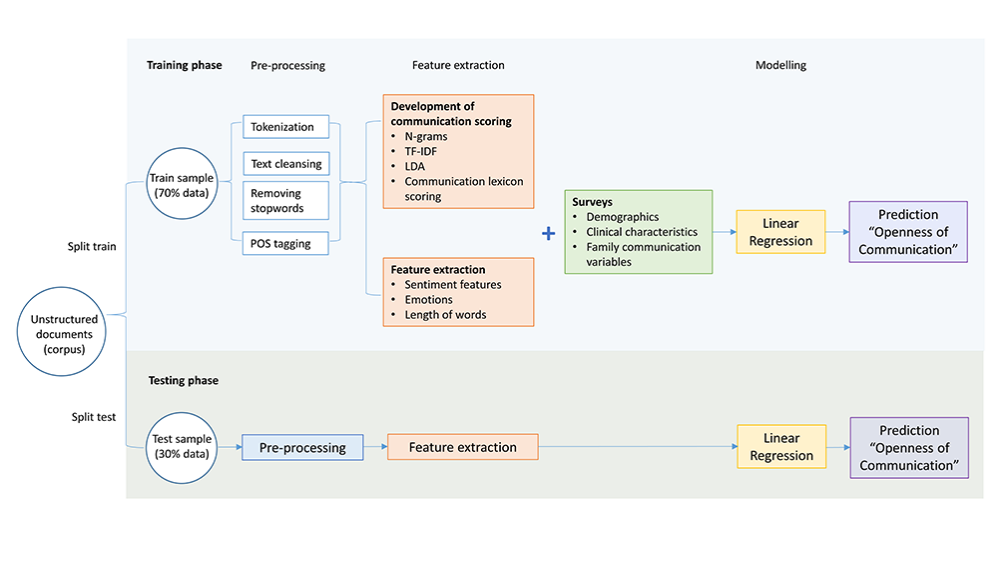
Phases of developing the natural language processing (NLP) algorithm: (1) preprocessing, (2) training, and (3) performance evaluation. LDA: latent Dirichlet analysis; POS: part of speech; TF-IDF: term frequency–inverse document frequency.

The ability of NLP to identify and predict different levels of “openness of communication” was evaluated following a multistep framework (Figure 1), which was divided into three phases: (1) preprocessing, (2) training, and (3) performance evaluation. All computations were performed in R software (version 3.6.3; R Foundation for Statistical Computing) [33]. We have made our analysis publicly available through the Zenodo open data repository [34].

### Preprocessing Phase

To start data processing, we broke down each text into individual tokens. We then applied functions to remove stop words and special characters. All texts were converted to lower case. We also applied part-of-speech tagging to extract phrases from the text corpus, used a latent Dirichlet allocation model to generate the most appropriate topics, and computed the term frequency–inverse document frequency to indicate the significance of a word in the text corpus [35,36].

#### Creation of a Lexicon and a Score for “Openness of Communication”

To develop an “openness of communication” score, we built a lexicon containing words and phrases linked to communication (for example, *“*difficulties in communication” and *“*excellent communication*”*) and classified them as positive or negative. After completing the preprocessing phase, we extracted N-grams from the text corpus. N-grams refer to single words (unigrams) or a combination of 2 or 3 words (bigrams or trigrams) associated with the outcome of interest, ie, “openness of communication.” To further enrich the lexicon, we applied the same process in a US-based sample of 123 narratives related to experiences with HBOC genetic cancer risk. This database includes narrative data collected between January 2013 to September 2016 from women and men who are carriers of HBOC-associated variants [26]. The semistructured interviews inquired about experiences with genetic counseling, genetic testing, and family communication patterns. We enriched the lexicon with supplementary words related to communication identified in an online thesaurus [37]. The final lexicon we created contained 532 items (132 unigrams, 215 bigrams, and 185 trigrams). Two members of the research team independently created the scoring of N-grams in the lexicon as positive or negative without considering the context of the phrases in the interviews. Specifically, they evaluated each item on a 7-point scale on how favorable the items measure “openness of communication.” Scoring values ranged from –3 (extremely strong negative word related to communication) to +3 (extremely strong positive word related to communication). In cases of disagreement, the final value was calculated by averaging the 2 values given by the 2 raters rounding to the greater nearest integer. The final “openness of communication” score assigned to the transcript of each narrative was developed by matching N-grams to the lexicon and summing up the corresponding scores. To ensure the robustness of the above scoring process, we calculated the Pearson correlation coefficient between the “openness of communication” scores we created with the IRI overall score. This correlation was examined only on Swiss data because Korean IRI scores were not available at the time of this analysis.

#### Sentiment Analysis and Attitude Toward Family Communication of Genetic Risk

To categorize the text of each narrative as describing either a positive or negative attitude toward genetic testing and health care services and to capture the overall emotional valence of the narrative, we used 3 common lexicons for text sentiment analysis: AFINN, Bing Liu, and the National Research Council Canada (NRC) Emotion Lexicon. The AFINN lexicon contains words with a score between –5 and +5, with negative and positive scores indicating negative and positive sentiments, respectively [38]. The Bing Liu lexicon classifies words into conveying a positive or a negative sentiment [1]. The NRC Emotion Lexicon estimates a sentiment score (positive and negative sentiment) based on 8 emotions. Positive emotions include anticipation, joy, surprise, and trust, whereas negative emotions include anger, disgust, fear, and sadness [39,40]. We also calculated an overall net sentiment expressed in each narrative, based on the difference between overall positive sentiment minus overall negative sentiment. An overall positive score meant that the individual expressed more positive sentiment in the narrative than negative, and vice versa.

### Training Phase

For developing the model, the overall data set was split randomly, with 70% of data used in the training phase by using the “openness of communication” score as the dependent variable. To examine whether the demographic and clinical characteristics and sentiment features of each narrative predicted “openness of communication” scores, we used a linear regression model based on the following steps. Initially we performed a univariate analysis to identify those independent variables exhibiting more than 60% absolute correlation with one another. These variables were excluded to avoid multicollinearity. Then, we continued with a multivariate analysis using a stepwise linear regression to identify possible predictors of the dependent variable and remove nonsignificant independent variables. As an alternative model, we attempted to use an artificial neural network. We built a fully connected network with 1 hidden layer, 1 input and 1 output layer, and 5 neurons. Optimization was done through the Broyden-Fletcher-Goldfarb-Shanno method. Early stopping was utilized to avoid overfitting. However, we ended up discarding the artificial neural network from the analysis because it showed no improvement compared to the linear regression. Finally, the performance of the models was evaluated using the area under the curve (AUC), sensitivity, specificity, and root mean square error (RMSE).

### Testing Phase

In this phase, we tested the model using the remaining 30% of the database (validation cohort). The performance of the models was evaluated using the same metrics as in the training phase, ie, AUC, sensitivity, specificity, and RMSE.

## Results

### Description of the Sample

Narrative and survey data from 53 individuals are included in this paper. Participants were aged 32-76 years. Most were female (47/53, 89%), married (41/53, 77%), and carriers of the familial pathogenic variant (51/53, 96%). Approximately 2 in 3 (32/53, 60%) had a prior diagnosis of cancer (Table 1). The Swiss and the Korean samples were not statistically different in respect to age (*P*=.71), prior cancer diagnosis (*P*=.38), educational level (*P*=.17), and employment status (*P*=.14).

**Table 1:** Sociodemographic and clinical data of participants (N=53).

Characteristic			Value
Age (years), mean (SD)			53.3 (12.1)
**Sex, n (%)**			
	Female	47 (89)	
**Education, n (%)**			
	Attended elementary/high school	5 (9)	
	High school graduate	14 (26)	
	Technical school graduate	13 (24)	
	University degree/postgraduate degree	21 (40)	
**Marital status, n (%)**			
	Married/living as married	41 (77)	
	Single	4 (8)	
	Divorced/separated/widowed	8 (15)	
Employed full or part time (yes), n (%)			34 (64)
**Cancer status, n (%)**			
	Previous cancer, one or more diagnoses	32 (60)	
	Never been diagnosed with cancer	21 (40)	
**Genetic test result, n (%)**			
	Positive for the familial pathogenic variant	51 (96)	
	Negative for the familial pathogenic variant	2 (4)	

### Description of the “Openness of Communication” Score and the Narrative Data

**Figure 2:**
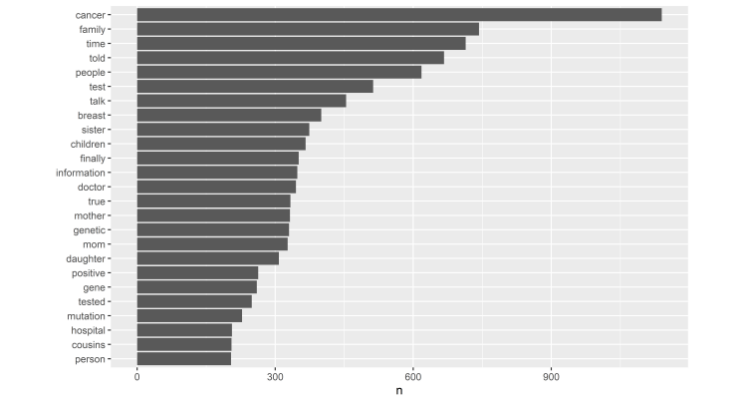
The most frequent words identified in narratives.

The average “openness of communication” score was 29.8 (SD 19.5; range –9 to 76), indicating an overall trend toward open communication. Narratives from these 53 individuals included 5837 unique unigrams, 4183 bigrams, and 654 trigrams. The most frequently appearing nontrivial words are shown in Figure 2. Based on the NRC Emotion Lexicon, the 10 most common positive words were “time,” “true,” “children,” “talk,” “finally,” “information,” “positive,” “doctor,” “understand,” and “daughter”. The 10 most common negative words were “cancer,” “sick,” “feel,” “risk,” “negative,” “died,” “difficult,” “fear,” “disease,” and “bad.”

### Validating the Relationship of “Openness of Communication” scores with IRI

The correlation coefficient between the “openness of communication” score and IRI in the Swiss data was *r*=0.46, indicating a moderate positive correlation.

### Attitude Toward Genetic Testing and Health Care Services

**Figure 3:**
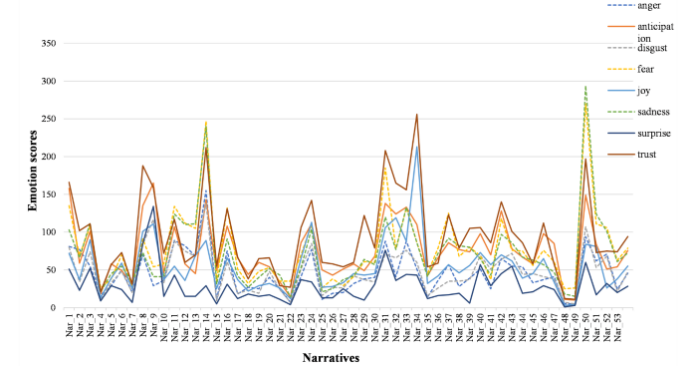
Frequencies of words identified for each emotion (anger, anticipation, disgust, fear, joy, sadness, surprise, and trust) based on the National Research Council Canada Emotion Lexicon.

Attitude toward genetic testing and health care services varied among participants, but it was overall positive. “Trust” appeared as the strongest positive emotion, whereas “fear” and “sadness” appeared as the strongest negative emotions in the text corpus based on the NRC Emotion Lexicon. The least perceived emotions were “surprise” and “anger.” Figure 3 describes the frequencies of words identified in the corpus for each emotion.

### Prediction of “Openness of Communication” Score

The *R*² for the overall model was 0.87 (adjusted *R*²=0.85; *P*<.001). A stepwise linear regression identified 5 significant predictors of “openness of communication” score, ie, the overall net sentiment of the narrative and fear, which were obtained based on the NRC Emotion Lexicon; informational support among family members; educational level; and being single (Table 2). Specifically, findings showed that both the higher overall net sentiment score of the narrative (*P*=.007) and also greater fear (*P=*1.97 × 10^–5^) were strongly associated with higher “openness of communication” scores. There was a positive correlation between “openness of communication” score and the statement “In our family when I have a health problem there is great willingness to share information with each other” (*P*=.005). Participants with nonacademic education were also more likely to communicate genetic risk with their relatives (*P*=.02). Lastly, there was a positive correlation between being single and “openness of communication” scores (*P*=.047).

**Table 2:** Results of the linear regression analysis predicting “openness of communication.”

Variables	Estimate	SE	*t* test^a^ (*df*)	*P* value
Being single	19.782	9.574	2.066 (1)	.047^b^
Academic education	–10.387	4.256	–2.44 (1)	.02^b^
Fear	0.204	0.041	4.954 (1)	1.97 × 10^–5c^
Informational support	11.392	3.790	3.006 (1)	.005^d^
Net sentiment score of the narrative	0.260	0.091	2.861 (1)	.007^d^

^a^2-tailed *t* test.

^b^Significance level: *P*<.05.

^c^Significance level: *P*<.001.

^d^Significance level: *P*<.01.

### Model Performance

**Figure 4:**
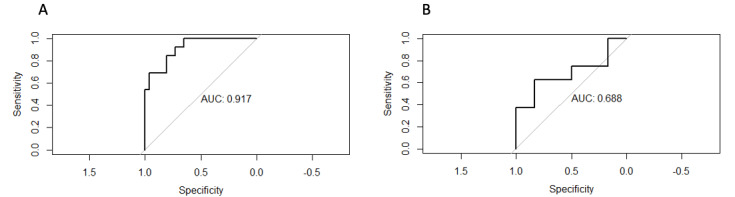
Receiver operating characteristic (ROC) curves of the training (A) and testing (B) model predicting “openness of communication” applying linear regression. AUC: area under the curve.

The predictive accuracy of the model using a stepwise linear regression for the training and testing data sets reached 0.85 (AUC=0.92, specificity=0.86, and sensitivity=0.82) and 0.72 (AUC=0.69, specificity=0.62, and sensitivity=0.83), respectively. Figure 4 presents the receiver operating characteristic curves that visualize the accuracy improvement between the training and testing data sets applying linear regression.

**Figure 5:**
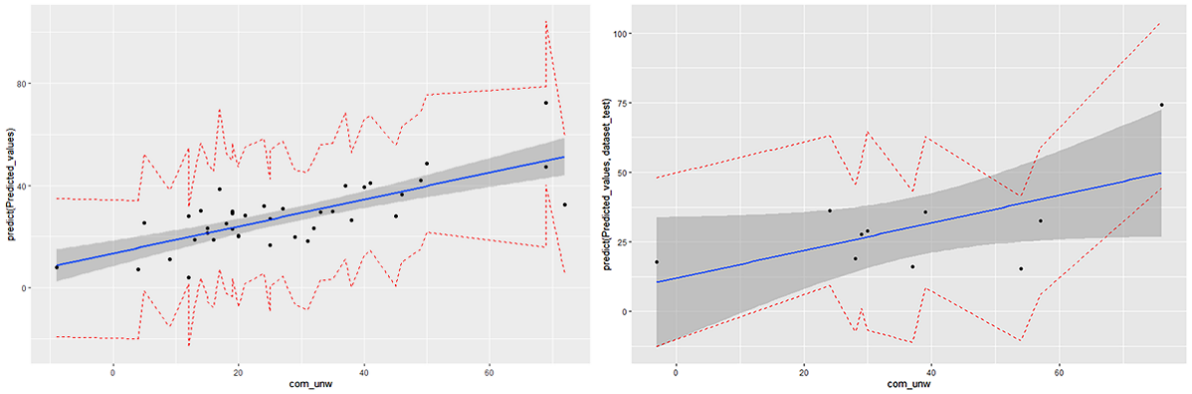
Scatter plot of predicted values against target values with 95% confidence and prediction intervals for the training (A) and testing (B) data sets applying linear regression.

The predicted values are plotted against the target values and are shown on a scatter plot for the linear regression model (Figure 5). The linear regression model achieved RMSEs of 11.76 (training data set) and 16.04 (testing data set). In this case, our model performs more accurately when it yields lower values of RSME.

## Discussion

### Principal Findings

We analyzed 53 narratives regarding intrafamilial communication of genetic cancer risk associated with HBOC. NLP enabled the analysis of unstructured narratives from different languages and identified the most frequently used words or combination of words describing openness in family communication of genetic cancer risk. This was the first study in which we applied NLP and sentiment analysis to better understand factors driving open intrafamilial communication regarding genetic cancer risk. Our findings showed that sentiment plays a crucial role and that emotions are a pervasive feature that predict intrafamilial communication in this particular population. Sentiment analysis performed on all interviews provided scores demonstrating positive or negative emotional valence, which were highly predictive of the direction of intrafamilial communication in this context. The higher overall net sentiment scores predicted greater openness in intrafamilial communication, whereas the lower overall net sentiment scores predicted closed or absent communication. This finding provides insights consistent with social penetration theory related to self-disclosure of carrying a cancer-causing genetic variant [41,42]. The depth of self-disclosure, ie, the degree to which the individual reveals personal and private information involving unusual traits and painful memories, reflects the degree of intimacy of a relationship. In the context of HBOC intrafamilial communication, self-disclosure of personal genetic information may be opposed by the desire to retain privacy and to avoid creating uncertainty and unpredictability in interpersonal relationships. Anticipating future negative emotions, such as regret or conflict, categorizes genetic risk information as a considerable emotional threat [43]. This finding was captured in our analysis as the overall net sentiment of each narrative, and its predictive value was confirmed based on the performance of our models. Taken together, findings indicate that sentiment can be used to frame genetic cancer risk as an opportunity for proactive risk reduction and for enhancing technology-mediated HBOC intrafamilial communication.

Our linear regression model explained more than 80% of the variance in openness of communication and achieved good performance in both the training and the testing samples. Our findings show that NLP was highly accurate in analyzing unstructured narratives from individuals of different cultural and linguistic backgrounds (Swiss German, French, Italian, English, and Korean) and in quantifying openness of communication in intrafamilial discussions about genetic cancer risk. The “openness of communication” scores were also validated against IRI. IRI was developed on the premise that increased genetic knowledge, positive motivation, and increased self-efficacy are prerequisites of increased intention to inform relatives about genetic risk. Although “intention to inform relatives” is closely related to “openness of communicating genetic risk,” the 2 concepts are not identical, which was also confirmed in our data with a moderate positive correlation between the 2 scores. An individual may have high intention to inform relatives about their genetic risk despite difficulties in communication within their family.

Creating a new lexicon for openness in communication enriched with terms from different sources contributes to the innovation of our approach and the generalizability and applicability of our findings. Our lexicon can be further used and expanded in future projects, providing a solid foundation for the use of NLP in the growing field of research in interpersonal communication, focusing on family communication and health care and technology-mediated communication [44]. Sentiment analysis can be further utilized in the era of precision medicine and precision public health for message tailoring and message framing. Extracting sentiment polarities can be highly informative in improving consumer experiences when using digital health platforms in promoting precision public health campaigns. For example, trust in the health care system has been associated with use of cancer surveillance, whereas conflicting messages from providers create a sense of disorientation and mistrust [45-48].

Our findings also indicated a greater likelihood of open intrafamilial communication in those who were single, had a nonacademic education, and higher informational support within their family network. These findings should be interpreted with caution and should be replicated with analyses of narratives from larger, and possibly more diverse, samples.

### Strengths and Limitations

Studies in different domains have also considered sentiment for analyzing textual communication in social media such as Twitter or Facebook [5,9,11]. However, one significant strength of our approach was that narrative data were combined with the demographic and clinical characteristics of participants, which can increase the applicability of findings. Another important strength was the use of several sentiment lexicons to select the most suitable for this context. Sentiment scores originating from the NRC Emotion Lexicon were the most appropriate to predict “openness of communication,” whereas the other 2 sentiment lexicons (AFINN and Bing Liu) were highly correlated, resulting in a predictive algorithm of lesser importance. Studies have shown that the selection of inappropriate lexicons may impact prediction performance [39,40]. Finally, NLP can automate parts of text analysis and can be used as an assisting tool to help researchers navigate through large volumes of text data.

One limitation of our study was the small sample size and the size of the available corpus, which did not allow us to include possible significant covariates and to fully explore the potential of the NLP methodology, including sentence structure and length of words. Despite this limitation, the results of our study can be used as indicators of various narrative features, such as overall sentiment and fear, which can be important predictors of interpersonal communication and self-disclosure in this specific population. Important features of NLP analysis, such as sentence structure and length of words, can be investigated with a larger number of narratives and larger number of corpora. The analytical approach we describe in this paper can be further improved by using larger samples. Further development of a robust model will advance a more precise assessment and reach higher accuracy.

### Conclusions

We demonstrate how various features from narratives can be used to predict “openness of communication” in individuals carrying a pathogenic variant connected to HBOC. Although our methodology requires further exploration and our findings require replication with larger samples, this is an important first step to understand how individuals and the public may react in discourses involving communication of genetic cancer risk. Overall, this experimental analysis provides evidence that our approach is promising and can be further used in the field of technology-mediated communication and precision public health.

## Abbreviations

AUC: area under the curve
HBOC: hereditary breast and ovarian cancer
IRI: Informing Relatives Inventory
LS: Lynch syndrome
NLP: natural language processing
NRC: National Research Council Canada
RMSE: root mean square error
